# 

**Published:** 1853

**Authors:** 


### INDEX TO VOL. XII.

A

Abscess of Kidney, recovery ........9
Agassiz, researches of............156
Anderson on regidity of os uteri...114
Association, Medical..............184
Anæsthetics in Dyspnœa..........231
Ascites, intra-peritoneal injections .266
Ascites, digitalis in................83
Air-compressor of testicle ........234
Asthma, Spasmodic ...............470
Acid Sulph. in cholera............475
Arteries, ligature of..............549
Amputations.....................550
Animal heat.....................313
Abscess, hepatic.................333
Anemia, fever, opium............340
Anesthetic, lycoperdon...........358

B

Bennet on the Uterus.............438
Bradford on Ovariotomy..........185
Branch on Veratrium viride.......120
Bronchitis, steam inhalation........76
Burdett on Typhoid Fever........369
Budd on the Liver................445
Boils...........................471
Bladder, puncture of.............552
Bladder, stone in.................291
Bartlett's case of Labor..........300

C

Cæsarian section..................74
Caldwell, memoir of..............101
Caldwell, tribute to..............177
Catalepsy, case of...............158
Cancer, statistics of Operations....232
Color-blindness..................170
Chapman, obituary of.............89
Clinical phrase-book..............30
Cold water reduction hernia........18
Cold water Phlegmasia dolens.....229
Chloroform..56, 99, 114, 474, 351, 353
Creosote.........................76
Clinical Midwifery................78
Cholera............274, 475, 520, 554
Claims of Medical profession......215
Cholera Infantum.................93
Coffee leaves for tea..............161
Catalepsy, remarkable case........158
Calculus, encysted...............164
Clark on Medical profession.......219
Consumption and Malaria.........227
Convulsions, epileptic............468
Carbuncle.......................471
Cataract........................283
Calculi from Stricture............289
Cochran, Dr., tribute to...........362

D

Darby on Dyspepsia..............278
Digitalis Fomentations.............83
Duty of Medical Men............174
Dysentery.......1, 490, 302, 337, 338
Double Monster...................61
Digestion.......................480
Dyspepsia with Hysteria..........278
Debow on a Sulphur Spring.......304
Dysmenorrhœa...................322

E

Elimination Elective..............65
Emmenagogues through mammæ..249
Ergot, substitute for.............332
Ergot in rigidity os uteri.........114
Ergot in premature Labor.........496
Ethnology.......................381
Eye, foreign body in.............286

F

Fatty Degeneration...............66
Fœtal Circulation.................75
Flint, Prof.................86, 337
Fever, Scarlet...................141
Fever, Typhoid..................369
Fever from decomposition.........221
Fishes, U. S., Agassiz............178
Fever, Intermit., common salt in..526
Fibrinous Deposits...............536
French Surgery..................283
Fracture-tables..................309
Fever, large doses quinine in......326
Fever, opium in.................340
Fever, Yellow, N. O.............355

G

Gresham on Hernia...............18
Gresham on Dysentery...........302
Gangrene.......................194

H

Hall, Dr. Marshall...............274
Headland, action medicines........20
Henle, general Pathology..........30
Hernia, cold water in..............18
Harris, lightning conductors.......39
Hæmorrhage from fatty degeneration 66

Health of Louisville..............85
Hydrophobia.....................82, 84
Heart, displacement of............188
Heart, wounds of................145
Hall on Metritis.................384
Head, impacted.................388
Harris, case of impacted head.....388
Herniotomy..... ............547
Hamilton's fracture-tables........309
Holmes' Poem...................323

I

Insanity.........................81
Iodine injections in dysentery.....338
Iodid. potas. in salivation.........347

K

Kidney, abscess of..................9
Kidney, diseases of.............. 491
Knee-joint, excision of.. .........559

L

Labor retarded by a tumor........300
Labor, its management...........496
Labor, Galvanism in .............508
Lipscomb on Moles.. ...........393
Lithotomy.......................547
Lightning-conducters..............39
Liver, Budd on..................445
Liver, abscess of.................333
Lycoperdon, anesthetic.... ........358

M

Malaria and Consumption.........227
Medicines, action of...............20
Mammæ, emmenagogues .........249
Meteorological Table..............92
Metritis........................384
Miller, W. H., case of Monstrosity.400
Moles, their origin...............393
Monstrosity, a case of............400
Medical Men, duration of life of....556
Medical Men, penalties of.........557
Medical Men, prosecutions of.....346
Mercury, iodide potas.............347
Microscope......................354

N

Neligan on skin diseases..........196
Nephritis, case of..................9
Nitrate of silver in tonsillitis......233

O

Ovariotomy ..........185, 49, 52, 272
Ovaritis........................228
Ox-gall.........................263
Os uteri, artificial dilatation of.....494
Operations in London Hospitals....547
Opium in Fever..................340

P

Pathology general ..................30
Phlegmasia dolens, cold water in....229
Poisoning by strychnine, chlorofrom 56
Placenta, fatty degeneration........66
Pneumonia, case of...............395
Pathology, surgical...............424
Paget's Lectures.................424
Psychotherapœia.................448
Parasites in disease............ ..320
Poem, Medical...................323

Q

Quinine in Rheumatism............72
Quinine, pills of.................154
Quinine, large doses in fever......326
Quicksilver, ring removal..........330

R

Rectum, through, for stone.......291
Rectum, stricture of..............294
Rheumatism ................72, 351
Ring removed by quicksilver......330
Rossey on Pneumonia............395

S

Salt, common, in intermittent fever 526
Scarlet fever....................141
Stomatitis materni...............175
Strychnine as a tonic..............70
Steam inhalations.................77
Stone in bladder..................87
Stone, hypogastrine operation.....294
Sneid, displacement heart.........188
Skin, diseases of.................196
Sulphuric acid in Cholera..........475
Sulphur spring in Tennessee......304
Spermatorrhœa..................335

T

Table-moving explained..........181
Testicle, air-compressor of........234
Trephining .....................549

U

Uva ursis.......................332
Urethra, stricture of..............289
Uterus, inflammation of..........438

V

Venereal diseases............... .401
Veratrum viride............120, 171
Vidal on Venereal diseases........401
Varicocele, Ricord's method.......298

W

Williams' French Surgery........283
Women, diseases of..............228

Y

Yandell, D. W. operations for stone..87
Yandell, L. P. on cholera infantum . 93
Yandell, L. P. memoir Dr. Caldwell 101
Yandell, L. P., case of abscess kidney 9
Yellow Fever in N. O............355

